# Application of indocyanine green-labeled fluorescence technology in laparoscopic total extra-peritoneal inguinal hernia repair surgery:a preliminary study

**DOI:** 10.1186/s12893-024-02505-0

**Published:** 2024-07-18

**Authors:** Qi Zhang, Xiujuan Xu, Jun Ma, Xinjian Ling, Yongsheng Wang, Yaming Zhang

**Affiliations:** 1 Department of General Surgery, Anqing Municipal Hospital, No. 352, Ren-Ming Road, Anqing, Anhui Province 246000 People’s Republic of China; 2 Department of Critical Medicine, Anqing Municipal Hospital, Anqing, 246000 People’s Republic of China

**Keywords:** Indocyanine green-labeled fluorescence laparoscopy, Spermatic artery, Inguinal hernia repair, Hemodynamic parameters, Complications, Bleeding

## Abstract

**Background:**

Laparoscopic Total Extra-peritoneal Inguinal Hernia Repair(TEP) presents escalated risks of surgical complications, notably bleeding, particularly in European Hernia Society (EHS) types 3 and recurrent inguinal hernia. In this study, we introduced an innovative technique using indocyanine green-labeled fluorescence laparoscopy to mitigate intraoperative complications, including bleeding and rupture of the hernial sac.

**Methods:**

This retrospective study reviewed records of 17 patients who underwent TEP repair at Anqing Municipal Hospital between July and August 2023. Intraoperatively, fluorescence imaging was utilized to trace the pathway of the spermatic vessels and outline the boundaries of the hernia sac to facilitate a thorough dissection.

**Results:**

The procedure was successfully completed in all 17 patients, with a median operation time of 42 min (range: 30–51 min). Median intraoperative blood loss was 5 ml (range: 3–8 ml). Complete dissection of the hernia sac was achieved in each case without any incidents of sac rupture. Hemodynamic parameters of blood flow within the spermatic artery on postoperative day 1 showed no statistically significant deviations from the preoperative values. Furthermore, during the 7-month follow-up period, there were no cases of seroma formation or hernia recurrence.

**Conclusion:**

Our findings suggest that employing indocyanine green-labeled fluorescence technology in TEP repair significantly reduces intraoperative complications, notably bleeding and rupture of the hernial sac. This technique demonstrated a negligible impact on the hemodynamic parameters of the spermatic artery and reduced the overall surgical time.

## Introduction

TEP repair, a tension-free hernia repair, has gained widespread acceptance in clinical settings due to its minimally invasive approach, comprehensive repair capability, low chronic pain incidence, and expedited postoperative recovery [1]. However, despite these advantages, TEP repair is not without risks, encompassing potential intraoperative complications such as bleeding, vascular and bladder injuries, intestinal damage, and nerve injury, as well as postoperative complications including seroma, infection, delayed wound healing, and intestinal obstruction. Notably, complications involving bleeding are comparatively more prevalent,the literature shows that the incidence of bleeding during TEP surgery is 3% -8%, but these studies did not differentiate inguinal hernia patients with different EHS subtypes [2, 3].

Prior research has indicated that in TEP repairs, the vessels of the spermatic cord are most susceptible to bleeding, followed by the corona mortis vessels and the venous vessels near the preperitoneal space of the pubic symphysis [4]. The spermatic cord predominantly comprises the internal and external spermatic arteries, the artery to the vas deferens, and the pampiniform plexus of veins. During laparoscopic IH repair, extraperitonealization of the spermatic cord is imperative. In patients with EHS types 3 IH, the formation of dense adhesions between the hernial sac and the spermatic cord at the internal ring poses significant surgical challenges. This complexity in the separation process heightens the risk of intraoperative damage to the spermatic cord tissues. In patients with recurrent IH, prior surgical interventions often compromise the space, resulting in scarring of the implanted patches and the spermatic cord tissues. This scenario increases the likelihood of collateral damage during the dissection process, consequently raising the risk of vascular collateral damage and potential vascular interruption. Following vascular injury, surgeons commonly resort to electrocoagulation or clamping for hemostasis. Although this technique effectively controls most bleeding, the collateral damage to the surrounding vessels remains unaddressed. This oversight can lead to various postoperative complications including ischemia and congestion of the testicle, testicular pain, and seroma formation, posing significant surgical challenges [5].

Fluorescence laparoscopy angiography has become widely used in clinical practice, particularly in hepatobiliary and gastrointestinal surgeries. Its applications include identifying tumor locations, assessing blood supply in anastomoses, and detecting lymph node metastases [6–8]. Yet, its adoption in IH surgery remains limited. In this study, we introduced an innovative technique using indocyanine green-labeled fluorescence laparoscopy to mitigate intraoperative complications, including bleeding and rupture of the hernial sac. This technique allowed vivid intraoperative visualization of the spermatic cord vessels and delineation of the boundary between the spermatic cord and hernial sac. This guidance is crucial in the intraoperative dissection of the hernia sac, potentially reducing both intraoperative and postoperative complication rates.

## Materials and methods

### Patients

We retrospectively reviewed records of 17 consecutive patients who underwent TEP repair at Anqing Municipal Hospital between July and August 2023. Only patients with IHs classified as EHS types 3 and recurrent were included in the study. Patients with a history of lower abdominal surgery,those with compromised cardiopulmonary function rendering them unsuitable for laparoscopic surgery and those with previous hypertension, hematological disorders, and long-term oral anticoagulant medication were excluded. We performed the procedures using the laparoscopic totally extra-peritoneal IH repair technique. All procedures followed were in accordance with the ethical standards of the responsible committee on human experimentation (institutional and national) and with the Declaration of Helsinki 1964 and later versions. The study protocol was approved by the Ethics Committee of Anqing Municipal Hospital. Informed consent was obtained from all participating patients.

All patients underwent standardized preoperative evaluations, including physical examinations, laboratory tests, and imaging studies. Key data such as age, sex, body mass index, hernia characteristics (primary or recurrent), herniation side (unilateral or bilateral), and specific surgical details, including operation time, blood loss, and hernia sac integrity, were meticulously recorded. Hemodynamic parameters of the spermatic artery were assessed via doppler ultrasound examination preoperatively and on postoperative day 1. Post-discharge follow-up was conducted to evaluate for postoperative complications such as seroma formation and hernia recurrence. Detailed patient characteristics are presented in Table 1.

**Table 1:** Patient characteristics

Variables	*N* = 17
Age (year)	61.4 ± 11.2
Sex	
Male	17
Female	0
Body mass index(kg/m2)	22.7 ± 3.5
Comorbidity	
Hypertension	0
Hematological system diseases	0
Anticoagulant therapy	0
Hernia	
Primary	14
Recurrence	3
Unilateral	15
Bilateral	2
Hemodynamics of the spermatic cord artery	
Peak systolic velocity (cm/s)	10. 17 ± 2. 02
End diastolic velocity (cm/s)	4. 04 ± 1. 12
Resistance index	0. 62 ± 0. 07

Values are presented with mean ± standard deviation or median (range)

## Methods

**Fig.1:**
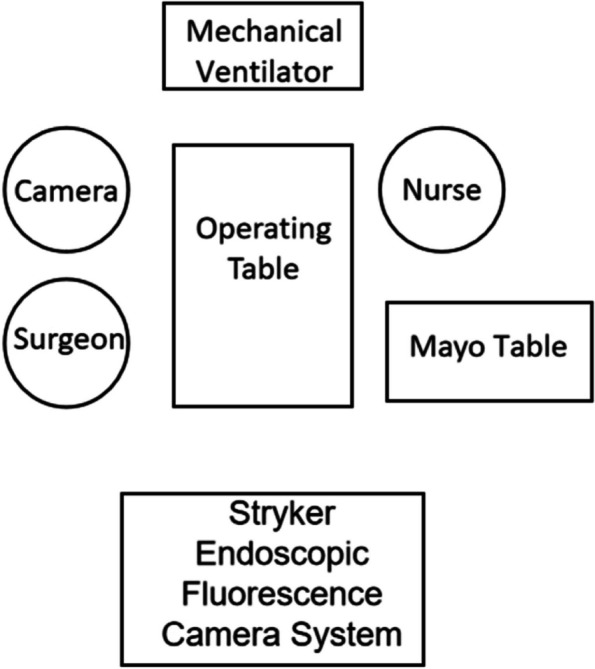
Layout of the surgical team, equipment, and operating table

**Fig. 2:**
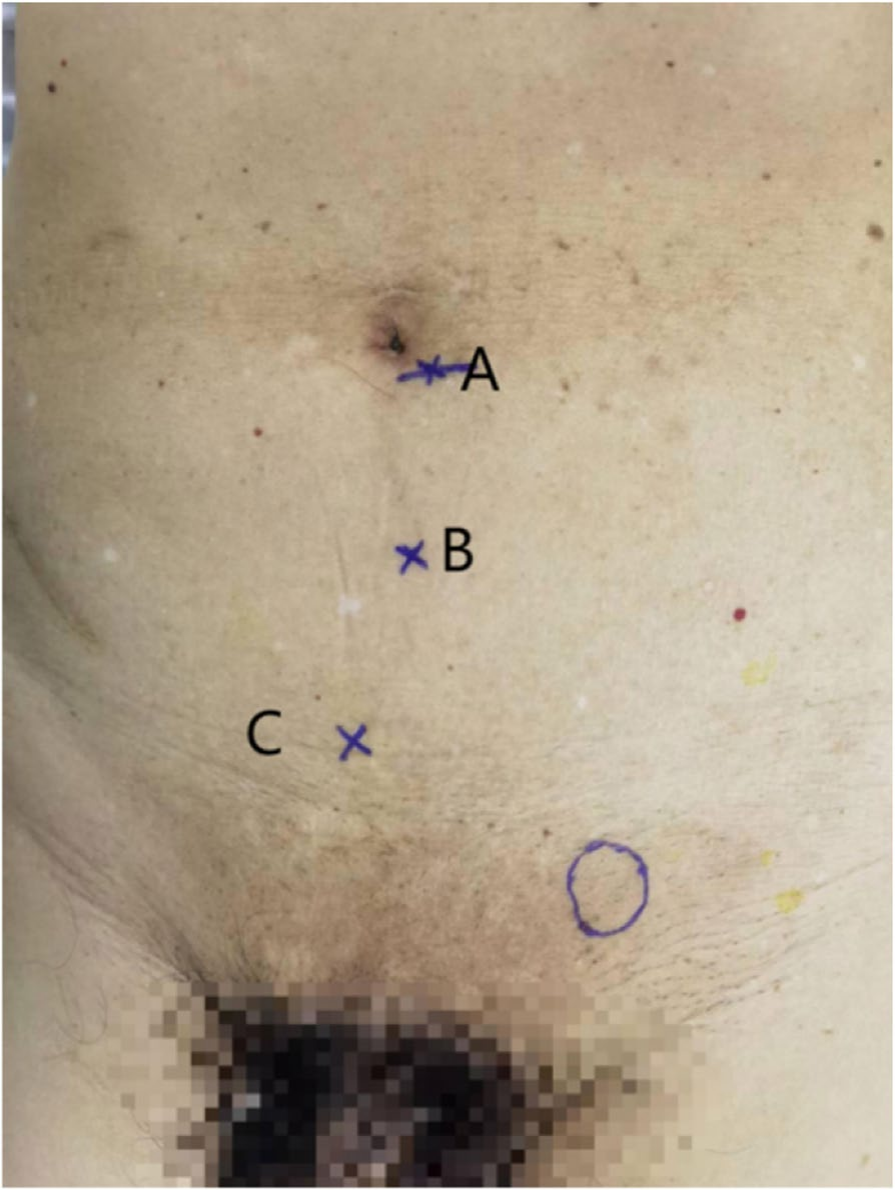
Layout of the trocar (**A** 12-mm trocar and **B** and **C** 5-mm trocar)

Upon general anesthesia administration, the patients were placed in a 15-degree Trendelenburg supine position. The surgeon’s positioning system and the setup of the laparoscopic system are detailed in Fig. 1. The surgical procedure adhered to the standard three-port technique conducted in a semi-lateral position, as illustrated in Fig. 2.

### Surgery

**Fig. 3:**
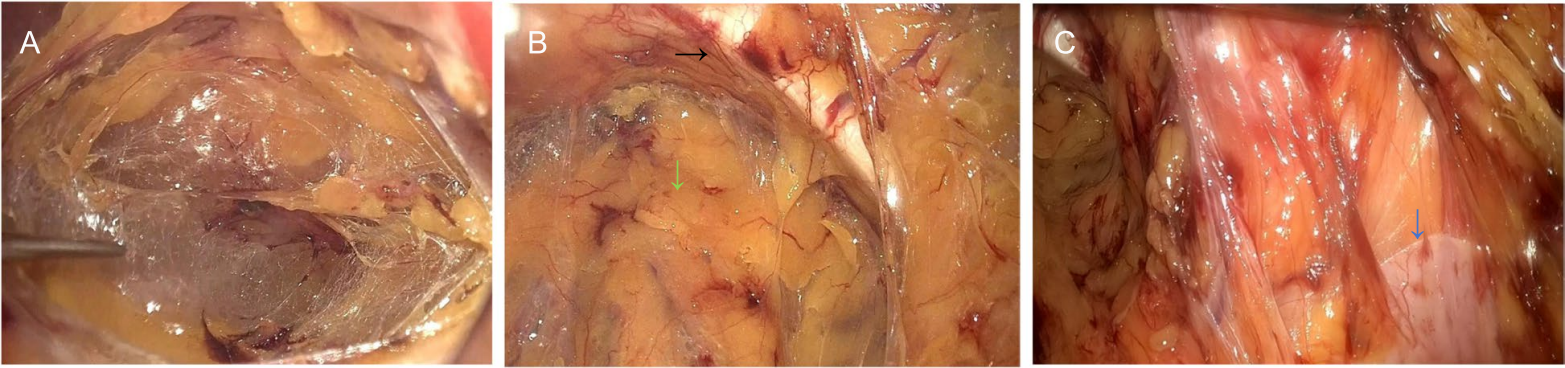
Surgical process. **A** Establish a preperitoneal space. **B** Dissection of the Retzius space. Pubic bone (black arrow) and bladder (green arrow). **C** Dissection of the Bogros space.The peritoneal reflection line(blue arrow)

Following general anesthesia administration, the surgeon made an approximate 12-mm arcuate incision below the patient’s navel, serving as an observation port (Fig. 2A). The anterior sheath of the rectus abdominis muscle was then incised and separated from the posterior sheath. A 12-mm trocar was subsequently inserted into the extraperitoneal space and securely positioned. A pneumoperitoneum machine was then connected to create a CO_2_ pneumoperitoneum within the abdominal cavity, maintaining a pressure of 11–13 mm Hg. The surgeon created two small 5-mm incisions: one positioned 3 cm below the observation hole (Fig. 2B), and the other 5 cm above the pubis and 2 cm lateral to the midline (Fig. 2C). These incisions served as operating ports into which a 5-mm trocar was inserted. First, the dissection of the Retzius space was performed, extending medially beyond the pubic symphysis, inferiorly beyond the pubic comb ligament by approximately 2 cm, and laterally approaching the vicinity of the inferior epigastric artery(Fig. 3A,B). Subsequently, with the inferior epigastric artery serving as a pivotal landmark, the procedure maneuvered over the top of the hernia sac to access the Bogros space. This was achieved under the guidance of the peritoneal reflection line(Fig. 3C). A comprehensive dissection of the Bogros space was then performed, extending to the medial boundary of the spermatic cord tissue and laterally to the projection of the anterior superior iliac spine.

**Fig. 4:**
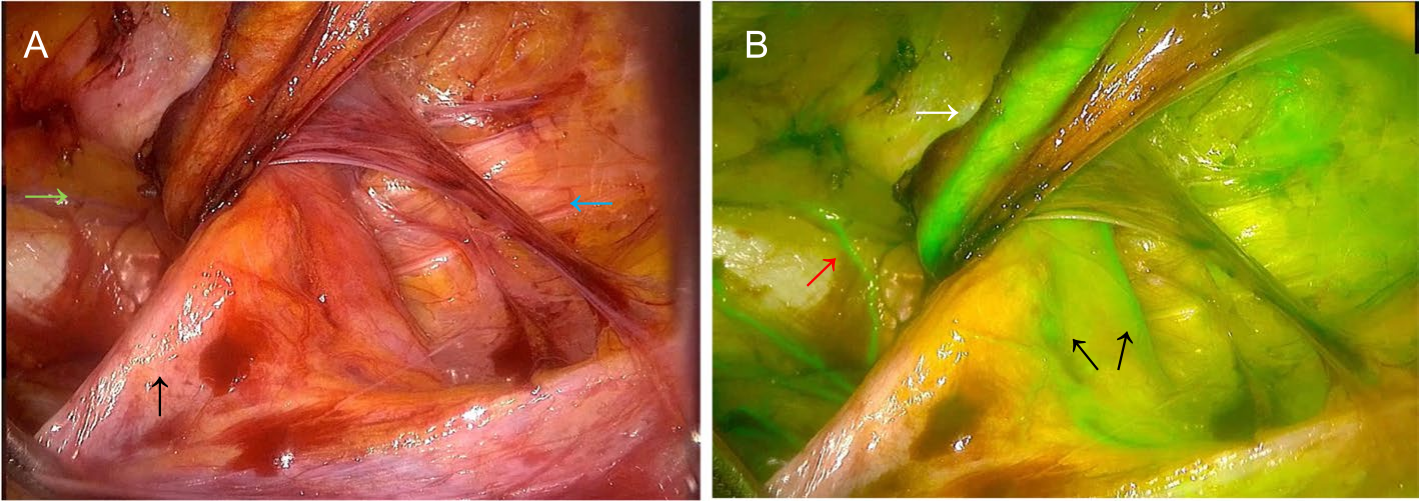
Comparison of myospectral origin in two modes. **A **Dissection of the Retzius(green arrow) and Bogros(blue arrow) spaces under conventional laparoscopic mode. The hernia sac was pulled medially(black arrow). **B ** Demarcation between the spermatic cord tissue and the hernia sac under fluorescence mode. The inferior epigastric artery(white arrow), the corona mortis(red arrow) and spermatic artery(black arrow) are visible

After the dissection of the bilateral spaces was finalized, the surgeon maneuvered the hernia sac medially. This action effectively spread the hernia sac and spermatic cord tissue, laying them flat at the center of the video screen (Fig. 4A). Subsequently, we diluted 25mg of indocyanine green solution in 10ml of physiological saline and rapidly injected it through peripheral veins, followed by switching the system to fluorescence mode. Approximately 30 s later, the visualization of the spermatic cord blood vessels, inferior epigastric artery, and corona mortis was achieved. This enhanced view delineated the separation of the hernia sac from the spermatic cord tissue. In the fluorescence mode, the surgeon observed the distribution of the spermatic cord vessels to accurately determine the boundaries of the hernia sac, facilitating complete sac dissection (Fig. 4B),simultaneously record intraoperative bleeding using 5 * 5cm gauze. Ultimately, a suitable mesh patch was selected to cover the openings in the muscle and pubic bone. Following this, the valve was opened to release the CO_2_ gas. The final step involved the closure of the surgical incisions.

## Results

### Surgical outcomes and postoperative complications

All 17 patients underwent successful surgery, with a median operation time of 42 min (range: 30–51 min) and a median intraoperative blood loss of 5 ml (range: 3–8 ml). Complete dissection of the hernial sac was achieved in all cases, with no instances of sac rupture. Hemodynamic parameters of the spermatic artery, reassessed on postoperative day 1, showed no statistically significant deviations from the preoperative values. Physical examinations conducted on postoperative day 3 revealed no scrotal effusion, testicular enlargement or pain, or local hematoma in any patient, facilitating a smooth discharge process. Detailed data are shown in Table 2.

**Table 2:** Surgical outcomes and postoperative complications

Variables	*N* = 17
Operation time (min)	42 (30–51)
Blood loss (ml)	5 (3–8)
Postoperative hospital stay (days)	3
Hernia sac damage	
Yes	0
No	17
Hemodynamics of the spermatic cord artery	
Scrotal hydrocele	
Yes	0
No	17
Testicular enlargement or pain	
Yes	0
No	17
Hematoma	
Yes	0
No	17
Hemodynamics of the spermatic cord artery	
Peak systolic velocity (cm/s)	10. 28 ± 2. 33
End diastolic velocity (cm/s)	4. 09 ± 1. 11
Resistance index	0. 61 ± 0. 08

Values presented with mean ± standard deviation or median (range)

### Postoperative follow-up

Of 17 patients, 16 were successfully followed up in an outpatient setting over a median period of 7 months(range: 6–8 months), while one patient was lost to follow-up. Among those who completed the follow-up, there were no reported cases of seroma formation nor any incidences of IH recurrence.Detailed data are shown in Table 3.

**Table 3:** Postoperative follow-up status

Complication (2-month follow-up)	*N* = 16
The median follow-up time(months)	7(6–8)
Seroma	
Yes	0
No	16
Hernia recurrence	
Yes	0
No	16

## Discussion

With advancements in laparoscopic technology, laparoscopic IH repair surgery is increasingly becoming the preferred approach. This method offers several advantages over open surgery, including reduced trauma and quicker postoperative recovery. However, the operating space in laparoscopic surgery is relatively limited compared to open surgery. Consequently, managing vascular injuries during laparoscopic procedures is extremely challenging, potentially leading to serious complications. The incidence of hematoma following laparoscopic IH repair surgery ranges from 3%–8% (average: approximately 3.4%). Factors such as chronic anticoagulation therapy, surgeries for recurrent hernias, mesh fixation, and the presence of large hernia defects reportedly increase the risk of hematoma formation [9–11]. Consequently, it is imperative to thoroughly recognize and vigilantly monitor for intraoperative bleeding, actively prevent it, and manage any bleeding incidents promptly and effectively.

Given that several major blood vessels are encountered during laparoscopic IH surgeries, we conducted a statistical analysis to identify the anatomical sites most susceptible to significant bleeding during these surgeries. The findings revealed that the vessels of the spermatic cord were most prone to bleeding, followed by the cover of the oblique hernia, area between the pubis and bladder, inferior epigastric vessels, and vessels of the vas deferens. The spermatic cord comprises the vas deferens and spermatic cord vessels. In the laparoscopic hernia repair visual field, the vas deferens is situated medially, whereas the testicular vessels are located laterally. During hernia sac separation, achieving “abdominalization of the spermatic cord” is essential [12]. In patients with EHS types 3 and recurrent IHs, the adhesion of the hernia sac and the presence of previously implanted mesh significantly increase the likelihood of collateral damage during the separation process [13]. Although intraoperative capillary bleeding from the hernia sac and tears in the spermatic cord vessels can be effectively managed with techniques such as electrocoagulation and clamping, these methods carry a significant risk of collateral damage. Reportedly, approximately one-third of patients may experience testicular atrophy following the disconnection of the spermatic cord in the inguinal canal; however, this seldom leads to testicular necrosis. Most cases manifest symptoms of ischemic testicular inflammation, including pain, swelling, and warmth in the scrotum and testicles, along with an elevated white blood cell count, which may eventually progress to testicular atrophy [14–16]. The clinical manifestations of such collateral damages are often concealed by compensatory collateral circulation. Nevertheless, these irreversible damages can impair reproductive function, increase the risk of hernia recurrence, and potentially necessitate a second surgical intervention.

In this study, following the dissection of the Retzius and Bogros spaces, the hernia sac was carefully retracted and spread along with the spermatic cord tissue within the surgical field. Subsequent intravenous injection of indocyanine green and the switch to fluorescence laparoscopy mode allowed for a vivid visualization of the branching and positioning of the spermatic artery. This enhanced view facilitated the easy recognition and separation of the hernia sac from the spermatic cord tissue, thus streamlining the dissection process of the hernia sac. The advantages of this surgical method may be due to: (1) Time efficiency: Despite the additional steps of intravenous injection and waiting for vascular imaging, the time required for hernia sac dissection is substantially reduced, thus decreasing the overall surgical time. (2) Reduced intraoperative blood loss: In patients with EHS types 3 and recurrent IHs conventional laparoscopic dissection often leads to misjudgment of hernia sac boundaries, resulting in inadvertent manipulation of vessels within the spermatic cord tissue and causing intraoperative bleeding. However, under fluorescence mode, the blood vessels of the spermatic cord, including capillaries on the hernia sac, are clearly visible. This allows surgeons to accurately predict the location and boundaries of vessels, significantly minimizing bleeding during surgery. (3) Avoidance of rupture of the hernial sac: The use of fluorescence mode distinctly highlights the blood vessels. Our observations indicated that due to the relatively lesser blood supply to the hernia sac tissue compared to the spermatic cord tissue, fluorescence mode revealed a clear line of separation between the hernia sac and the spermatic cord. This distinction greatly reduced the difficulty of hernia sac dissection during surgery. (4) Minimal impact on hemodynamic parameters of spermatic cord vessels: Laparoscopic IH surgery typically involves electrocoagulation and clamping for hemostasis of bleeding from the spermatic cord tissue. In addition, the hernia sac dissection process often necessitates repeated manipulation of the hernia sac and spermatic cord tissue, leading to collateral damage to the spermatic cord vessels. In contrast, the dissection process under fluorescence staining mode is simpler, requires fewer manipulations, and results in less bleeding. Postoperative color Doppler ultrasound examinations confirm that there are no significant changes in the hemodynamic parameters of the spermatic cord vessels.

## Conclusion

Our findings suggest that employing indocyanine green-labeled fluorescence laparoscopy in Nyhus types III and IV IH repairs significantly reduced intraoperative complications, notably bleeding and rupture of the hernial sac. This technique demonstrated a negligible impact on the hemodynamic parameters of the spermatic artery and reduced the overall surgical time. However, further studies with larger sample sizes and regular follow-ups are warranted to observe key variables, including postoperative recurrence, seroma formation, and long-term prognosis. Given the limitations inherent in the single-arm study design of our study, we plan to undertake future controlled studies to further ascertain the clinical value of fluorescence technology in IH repair.

## Data Availability

Data is provided within the manuscript or supplementary information files.

## Abbreviations

TEP: Laparoscopic total extra-peritoneal inguinal hernia repair
EHS: European Hernia Society
IH: Inguinal hernia
BMI: Body mass index
Fig: Figure
